# Effects of Dietary Calcium and Phosphorus Levels on Growth Performance, Calcium–Phosphorus Homeostasis, and Gut Microbiota in Ningxiang Pigs

**DOI:** 10.3390/life15071083

**Published:** 2025-07-09

**Authors:** Wenzhi Liu, Cheng Zhang, Xijie Kuang, Xianglin Zeng, Jiaqi Zhang, Qiye Wang, Huansheng Yang

**Affiliations:** 1 Hunan Provincial Key Laboratory of Animal Intestinal Function and Regulation, Hunan International Joint Laboratory of Animal Intestinal Ecology and Health, Laboratory of Animal Nutrition and Human Health, College of Life Sciences, Hunan Normal University, Changsha 410081, China; 202370142924@hunnu.edu.cn (W.L.);; 2 Key Laboratory of Agro-Ecological Processes in Subtropical Region, Hunan Provincial Engineering Research Center of Healthy Livestock, Scientific Observing and Experimental Station of Animal Nutrition and Feed Science in South-Central, Ministry of Agriculture, Institute of Subtropical Agriculture, Chinese Academy of Sciences, Changsha 410125, China

**Keywords:** calcium, phosphorus, bone mineralization, intestinal morphology, gut microbiota

## Abstract

Optimal dietary calcium (Ca) and phosphorus (P) requirements remain undetermined for Ningxiang pigs, a valuable indigenous Chinese breed. This study conducted a continuous feeding trial with two growth phases (grower: 30–50 kg; finisher: 50–80 kg) using fixed Ca/P ratios to systematically evaluate the effects of Ca/P levels on growth performance and mineral metabolism. A total of 180 pigs per phase were allocated to four Ca/P levels. During the grower phase, a dietary regimen of 0.83% Ca/0.67% P significantly increased the average daily feed intake (ADFI), average daily gain (ADG), and apparent total tract digestibility (ATTD) of energy and P. In the finisher phase, 0.60/0.48% Ca/P showed optimal growth performance, upregulated jejunal mineral transporters (*CaSR* and *SLC34A2*), enhanced bone mineralization (metatarsal ash content), and improved intestinal morphology (duodenal and jejunal villus height, jejunal villus surface area). This regimen also selectively enriched *Peptostreptococcaceae* abundance, indicating improved host–microbe interactions. Based on these findings, stage-specific nutritional strategies were recommended: 0.83% Ca/0.67% P during the grower phase and 0.60% Ca/0.48% P during the finisher phase. These protocols synergistically improve microbial ecology, intestinal function, and bone metabolism, thereby maximizing the growth potential of Ningxiang pigs.

## 1. Introduction

Calcium (Ca) and phosphorus (P), the most abundant mineral elements in pigs, are predominantly stored in bone tissue as hydroxyapatite [Ca_10_(PO_4_)_6_(OH)_2_] [1]. Beyond skeletal integrity, the remaining 1% of Ca, primarily as Ca^2+^ ions, participates in essential physiological processes, including muscle contraction, nerve signaling, and enzyme activation [2]. Similarly, phosphorus is vital for metabolic and signaling pathways and, synergistically with Ca, regulates bone metabolism and immune function [3]. Dietary Ca/P imbalances induce skeletal deformities and growth retardation, compromise intestinal integrity and morphology, and perturb the gut microbiota composition, thereby impairing growth performance [4,5,6].

Amidst growing consumer demand for premium pork, Ningxiang pigs—a genetically distinct Chinese indigenous breed prized for exceptional meat quality, disease resistance, and adaptability to coarse feed [7]—represent a valuable agricultural genetic resource. The precise dietary Ca and P requirements necessary to optimize growth performance in this specific breed remain undetermined. This gap is particularly relevant because Ningxiang pigs, characterized as a fat-type breed, exhibit distinct metabolic patterns favoring fat deposition and possess unique intestinal and metabolic microbial profiles compared to commercial crossbred pigs [8,9]. Consequently, understanding the relationship between their specific metabolism and mineral utilization is crucial. Optimizing the Ca/P balance is vital not only for growth metrics like average daily gain (ADG) and feed efficiency [5] but also for enhancing intestinal nutrient absorption capacity through improved morphology [7]. The lack of breed-specific data hinders efficient feeding management and production optimization in Ningxiang pigs.

Therefore, to determine the optimal Ca and P requirements for Ningxiang pigs, this study referred to the recommendations outlined in GB/T 39235-2020 for fattening pigs, as well as to previous research [8,10]. Diets with graded levels of Ca and P were formulated at a fixed Ca/P ratio of 1.24:1. This study systematically evaluated these diets during two key growth stages: the growing phase (30–50 kg body weight) and the finishing phase (50–80 kg body weight). The evaluation encompassed multiple parameters, including growth performance, skeletal characteristics, serum biochemistry, intestinal morphology, apparent total tract digestibility (ATTD), mRNA expression of Ca and P transport proteins (CaSR, SLC34A2), and gut microbiota composition. The primary objective was to determine the Ca and P levels that maximize growth potential in Ningxiang pigs, while also investigating their impact on metabolic status and microbiota. This research aims to provide a scientific basis for breed-specific feeding strategies and potentially reduce environmental mineral excretion.

## 2. Materials and Methods

### 2.1. Animal Welfare Statement

All animal experimental procedures were conducted in strict accordance with protocols approved by the Animal Care and Use Committee of Hunan Normal University (Approval number: 2023-209).

### 2.2. Animals, Diets, Treatments, and Feeding Management

**Figure 1:**
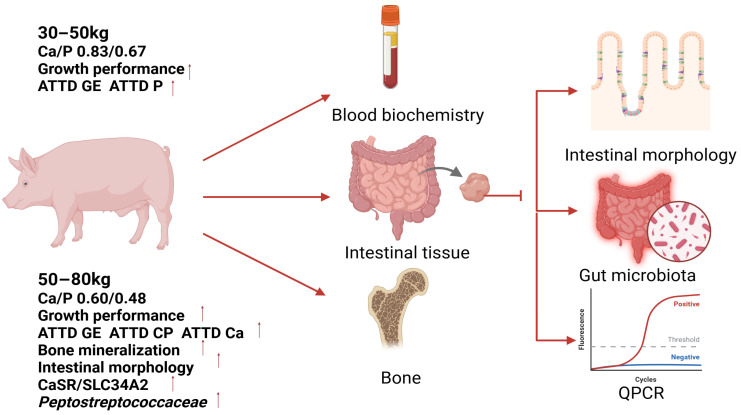
Experimental design flowchart. “
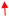
”, upregulated.

This study employed a two-phase experimental design reflecting commercial production practices: a growth phase (30–50 kg body weight) followed by a fattening phase (50–80 kg body weight). For the growth phase (Phase I), 180 Ningxiang pigs with an initial weight of 32.28 ± 1.78 kg were randomly assigned to four dietary treatments (A1, B1, C1, or D1), each comprising five replicates of nine pigs per replicate. Over a period of 41 days, these groups received diets with graded Ca/P levels: 0.52/0.42% (A1), 0.62/0.50% (B1), 0.73/0.59% (C1), and 0.83/0.67% (D1). Upon completion of the growth phase, 180 Ningxiang pigs averaging 50.93 ± 2.49 kg entered the fattening phase (Phase II), using an identical replication structure (five replicates per treatment, nine pigs per replicate). During this 56-day period, the dietary treatments consisted of 0.50/0.40% (A), 0.60/0.48% (B), 0.70/0.56% (C), and 0.80/0.64% (D) Ca/P levels. All of the experimental diets were formulated in accordance with GB/T 39235-2020 “Nutritional Requirements for Pigs” for fat-type breeds [10], with detailed formulations and nutritional compositions provided in Table 1 (growth phase) and Table 2 (fattening phase). The experimental design is illustrated in the flowchart presented in Figure 1.

The experiments were conducted at the breeding base of Hunan Chuwei Xiang Agricultural and Livestock Co., Ltd. (Changsha, China). In the experiments, each dietary treatment group included five pens, with each pen (4.0 × 8.0 m) housing nine pigs. The number of castrated boars and sows was kept consistent across all groups. Throughout the experimental period, pigs had ad libitum access to both feed and water. Daily records were maintained for feed intake and the health status of each experimental pen. The enclosures were cleaned daily to ensure hygiene. Throughout the rearing period of the experimental pigs, feeding protocols, daily management, and routine vaccination procedures were strictly adhered to, in accordance with the company’s guidelines.

### 2.3. Sample Collection

In the last three days of both experiments, fecal samples were collected daily from each pig in each pen using the total feces collection method, during four specific time intervals: 09:00–09:30, 12:00–12:30, 17:00–17:30, and 21:30–22:00. After collection, the fecal samples from each pen were thoroughly mixed in equal proportions and divided into three aliquots (each ≥500 g). Concurrently, corresponding feed samples were collected from daily from each pen. The collected feces and feed samples were pooled, homogenized, and stored at −20 °C for subsequent analysis. During both experimental phases, the daily feed intake of each pig was measured to determine their daily consumption. At the conclusion of the experiment, the pigs were weighed to evaluate the growth performance of Ningxiang pigs. The ADFI, ADG, and feed-to-gain ratio (F:G) were carefully calculated for each phase [11].

After a 12 h fasting period, eight pigs whose body weights were closest to the average of their respective treatment group were selected. Blood samples (5 mL) were drawn from the anterior vena cava via catheterization and placed into heparinized tubes. The samples were centrifuged at 4000 rpm for 10 min, and the resulting supernatant was transferred into sterile 1.5 mL Eppendorf tubes before being stored at ultra-low temperatures for future analysis.

Following the completion of the fattening phase, eight pigs from each group (ensuring each replicate included at least one pig), selected based on average body weight, were slaughtered. Samples from the right hind tibia and fourth toe bones were collected, weighed, and stored at −20 °C. Segments of the duodenum (10 cm, located 15 cm distal to the pyloric sphincter) and ileum (10 cm, proximal to the ileocecal valve) were dissected, longitudinally opened, and gently washed with tap water to remove the residual contents. Duodenum and jejunum samples were carefully washed with saline and preserved in 10% neutral buffered formalin for subsequent histological analysis of the intestinal structure. Equal portions of duodenum and jejunum samples were snap-frozen in liquid nitrogen for future molecular biological analyses.

### 2.4. Measurements of Serum Parameters

The biochemical parameters of Ningxiang pig blood were analyzed using an automated biochemical analyzer (Cobas C311, Roche, Switzerland) at the Institute of Subtropical Agriculture, Chinese Academy of Sciences. The parameters evaluated included alkaline phosphatase (ALP), blood urea nitrogen (BUN), serum calcium (Ca), phosphorus (P), total cholesterol (CHOL), triglycerides (TG), and non-esterified fatty acids (NEFA). Pre-analytical quality control was conducted for all assays to ensure accuracy and reliability. The sample preparation and testing procedures strictly followed the operational guidelines provided by the automated biochemical analyzer’s manufacturer, with reagents supplied by Nanjing Jiancheng Bioengineering Institute (Nanjing, China).

### 2.5. Determination of Apparent Digestibility

The feces and feed samples were dried at 65 °C for 72 h until completely dry, after which the samples were ground into a fine powder. In accordance with the GB/T 23742-2009 standard [12], the acid-insoluble ash content in both the feed and feces was determined. Acid-insoluble ash was used as an internal, non-digestible marker to calculate digestibility. Ca levels were measured using the EDTA titration method (GB 5009.92-2016) [13], while P content was quantified via spectrophotometry (GB/T 6437-2018) [14]. Crude protein (CP) was analyzed using the Kjeldahl method (GB/T 6432-2018) [15]. The energy content of both feed and feces was assessed using an automatic precision intelligent calorimeter (MP-C(X)2000, Changsha, China). The apparent total tract digestibility (ATTD) of a specific nutrient in the diet was calculated using the following formula: $AIA\left(\%\right)=100-(\frac{M_{2n}}{M_{1n}}\times \frac{M_{1m}}{M_{2m}})\times 100$. In this context, AIA represents the nutrient digestibility rate (%), M_1m_ denotes the indicator content in the diet (g/g), M_2m_ refers to the indicator content in the feces (g/g), M_1n_ indicates the nutrient content in the diet (g/g), and M_2n_ denotes the nutrient content in the feces (g/g).

### 2.6. Measurement of Bone Characteristics

The fourth metatarsal was thawed at room temperature and meticulously cleaned of all adhering muscle and fascia using surgical scissors and a scalpel. The sample was subsequently placed in a muffle furnace and subjected to ashing at 850 °C for 15 h. Once cooled to room temperature, the ash was collected for the analysis of Ca and P contents. The tibia was removed from a −20 °C freezer and allowed to thaw naturally at room temperature. Upon complete thawing, its length was measured using a caliper. Adhering muscle, fascia, and other tissues were meticulously excised using surgical scissors and a scalpel on a clean workbench, after which the tibia was then weighed. In this study, the bone mineral density (BMD) of the tibiae of Ningxiang pigs was assessed using dual-energy X-ray absorptiometry (DXA) with a Hitachi DCS-600EXV scanner (ALOKA, Tokyo, Japan) at the Changsha Economic and Technological Development Zone Hospital.

### 2.7. Histomorphological Examination of Intestinal Tissue

Intestinal samples were initially removed from the fixative solution and rinsed with running water before being transferred to disposable embedding molds. The duodenum and jejunum samples underwent dehydration through a graded series of ethanol solutions, followed by immersion in xylene until complete transparency was achieved. Once fully transparent, the tissues were embedded in low-melting-point paraffin. After paraffin embedding, sections were prepared and stained with hematoxylin and eosin (H&E) [16]. The H&E-stained sections of the duodenum and jejunum from Ningxiang pigs were examined under a light microscope (DM3000; Leica, Wetzlar, Germany) to evaluate their morphological structure. Villus height (VH) and crypt depth (CD) were measured using Image-Pro Plus 6.0 software, with the villus surface area and VH: CD ratio subsequently calculated. For each sample, at least 30 intact villus–crypt units were randomly selected for analysis [17].

### 2.8. RNA Extraction and Real-Time Quantitative PCR Analysis

Gene expression analysis of intestinal calcium and phosphorus transporters in Ningxiang pigs was conducted using quantitative real-time PCR (qPCR) [18]. Briefly, total RNA was extracted from duodenal and jejunal tissues using RNAiso Plus reagent (TaKaRa, Dalian, China) and subsequently reverse-transcribed into complementary DNA (cDNA) with a PrimeScript RT reagent kit (TKR-RR047A, TaKaRa). The cDNA was dilution and used as a template for qPCR reactions performed on a QuantStudio 5 Real-Time PCR System (Thermo Fisher Scientific, Singapore) under the following parameters: 95 °C for 30 s, followed by 40 cycles of 95 °C for 3 s and 60 °C for 30 s. Each 10 μL reaction mixture contained 5.0 μL of SYBR Premix Ex Taq (TKR-RR0820A), 1 μL of diluted cDNA template, 0.2 μL each of forward/reverse primers (sequences provided in Table 3), 0.2 μL of ROX reference dye, and 3.4 μL of PCR-grade water. The ribosomal protein L4 (*RPL4*) gene served as an endogenous control for normalization. Relative mRNA expression levels were calculated using the 2^−ΔΔCt^ method, where ΔCt = Ct (target gene) − Ct (RPL4). All reactions were performed in triplicate to ensure technical reproducibility.

### 2.9. Analysis of Gut Microbiota Diversity

To investigate the relationship between dietary Ca/P levels and microbial ecology, full-length 16S rDNA sequencing was performed on colonic digesta samples collected from Ningxiang pigs. The sequencing was carried out by Shanghai Meiji Biomedical Technology Co., Ltd. (Shanghai, China) using the PacBio Sequel IIe platform. For bioinformatics analysis, the Majorbio cloud platform (available at https://www.majorbio.com/) was primarily utilized. Raw sequencing reads were processed using SMRT Link analysis software (version 8.0), with stringent quality control parameters (minimum of three full passes, ≥99% base-calling accuracy), to generate circular consensus sequencing (CCS) reads. Qualified CCS reads (1000–1800 bp) were demultiplexed based on sample-specific barcodes and filtered by length. Subsequently, sequences were clustered into operational taxonomic units (OTUs) at a 97% similarity threshold using the UPARSE pipeline (v7.1) [19]. The most abundant sequence within each OTU was designated as the representative sequence for taxonomic assignment. To standardize the number of sequences across all samples, subsampling was performed, followed by α-diversity analyses. Chao1 richness and Shannon/Simpson diversity indices were calculated using mothur (v1.45.1) [20], with Wilcoxon rank-sum tests conducted for group comparisons. The similarity of microbial community structures among samples was evaluated using partial least squares discriminant analysis (PLS-DA) based on Bray–Curtis distance matrices. Significant differences in microbial community structures among groups were assessed using PERMANOVA, a non-parametric method [21]. Additionally, we applied the linear discriminant analysis effect size (LEfSe) method (LDA score > 2, *p* < 0.05) to identify differentially abundant taxa across the phylum to genus levels.

### 2.10. Data Analysis

Data were presented as the mean ± standard error of the mean (SEM) for each group. The data were initially summarized using Excel 2019 (Microsoft, Redmond, WA, USA) and subsequently analyzed via one-way ANOVA using SPSS 20.0 (IBM, Armonk, NY, USA). Prior to conducting ANOVA, the data were tested for normality and homogeneity of variances. The number of replicates used for the statistical analysis of growth performance and gut microbiota indicators was set at 5 (n = 5), while the sample size for all other analyses was 8 (n = 8). For datasets that violated these assumptions, non-parametric tests were employed to calculate the *p*-values, thereby minimizing the risk of false positives and false negatives. The *p*-values for linear, quadratic, and overall trends were derived through curve estimation in regression analysis. A *p*-value ≤ 0.05 was considered statistically significant, 0.05 < *p* ≤ 0.10 indicated a trend, and *p* > 0.10 denoted no significant difference.

## 3. Results

### 3.1. Growth Performance

The effects of dietary Ca/P levels on the growth performance of Ningxiang pigs are summarized in Table 4 and Table 5. Table 4 indicates that, in the growth phase, increasing dietary Ca/P levels significantly enhanced ADFI (linear, *p* = 0.047) and exhibited a trend towards linear improvement in ADG (linear, *p* = 0.076). No significant effect was observed on F:G (*p* > 0.05). The optimal ADG and ADFI were achieved at a dietary Ca/P level of 0.83%/0.67%. According to Table 5, in the fattening phase, as the dietary Ca/P level increased, the highest ADG (*p* = 0.021) was recorded at a Ca/P content of 0.60%/0.48%, while no statistically significant differences were observed in ADFI and F:G (*p* > 0.05).

### 3.2. Serum Biochemical Indicators and Mineral Contents

As shown in Table 6, different levels of dietary Ca/P in the growth phase did not significantly affect the ALP, BUN, CHOL, TG, NEFA, serum Ca, or serum P concentrations in Ningxiang pigs (*p* > 0.05). In the fattening stage (Table 7), increasing dietary Ca/P levels had notable effects on certain serum parameters. Specifically, NEFA increased linearly with higher Ca/P intake (linear, *p* = 0.046). Serum Ca exhibited a linear decreasing trend, although this was not statistically significant (linear, *p* = 0.063). TG showed a quadratic trend, initially increasing and then decreasing, with the peak occurring at the 0.60/0.48% dietary Ca/P level (quadratic, *p* = 0.057). No significant effects were observed on ALP, BUN, CHOL, and serum P (*p* > 0.05).

### 3.3. Total Gastrointestinal Apparent Digestibility

Table 8 demonstrates that different dietary Ca/P levels significantly influenced the apparent total tract digestibility (ATTD) of gross energy (GE) and P in Ningxiang pigs during the 30–50 kg growth stage (*p* < 0.05). The highest ATTD values for GE and P were observed at a Ca/P level of 0.83/0.67%. Dietary Ca/P levels had no significant effect on the ATTD of Ca and CP (*p* > 0.05).

As shown in Table 9, during the 50–80 kg growth stage in Ningxiang pigs, the ATDD of GE exhibited a significant quadratic trend, initially increasing and then decreasing with elevated dietary Ca/P levels (*p* = 0.009; quadratic, *p* = 0.003), and peaking at the 0.60/0.48% level. Similarly, the ATDD of CP showed a quadratic pattern (quadratic, *p* = 0.012), also peaking at the 0.60/0.48% level. The ATDD of Ca followed a similar trend (*p* = 0.019; quadratic, *p* = 0.012), reaching its maximum at the 0.70/0.56% level. In contrast, no significant effect was observed on the ATDD of P (*p* > 0.05).

### 3.4. Bone Characteristics

**Figure 2:**
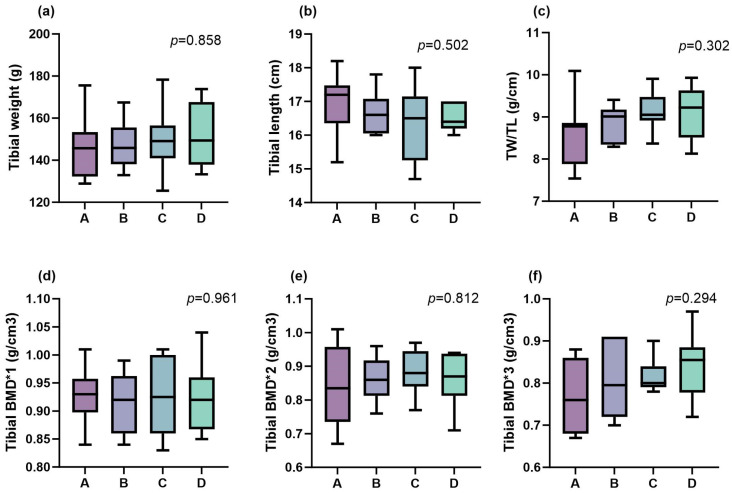
Effects of dietary calcium (Ca) and phosphorus (P) levels on tibial skeletal characteristics in Ningxiang pigs during the 50–80 kg period (n = 8): (**a**) tibial weight; (**b**) tibial length; (**c**) TW/TL ratio; (**d**) 1/3 Tibial BMD; (**e**) 1/6 Tibial BMD; (**f**) 1/10 Tibial BMD. Treatment groups: A: Ca/P level = 0.50/0.40; B: Ca/P level = 0.60/0.48; C: Ca/P level = 0.70/0.56; D: Ca/P level = 0.80/0.64. Tibial BMD^1*2*3^ indicates bone mineral density measured at the distal 1/3, 1/6, and 1/10 segments of the tibia, respectively. BMD: bone mineral density; TW: tibial weight; TL: tibial length; TW/TL: tibial weight per unit length; “*p*” represents the *p*-value.

**Figure 3:**
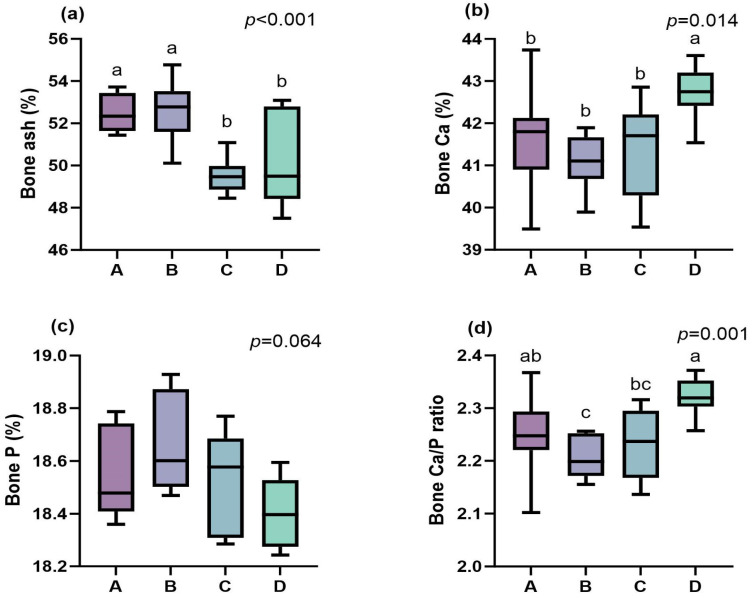
Effects of dietary calcium (Ca) and phosphorus (P) levels on the ash content (**a**), calcium content (**b**), phosphorus content (**c**), and the Ca/P ratio (**d**) in bone ash of the fourth metatarsal bone from Ningxiang pigs during the 50–80 kg fattening phase (n = 8). A: Ca/P level = 0.50/0.40; B: Ca/P level = 0.60/0.48; C: Ca/P level = 0.70/0.56; D: Ca/P level = 0.80/0.64. Different letters a, b, and c above the boxplot frame indicate statistically significant differences among different groups; “*p*” represents the *p*-value.

As shown in Figure 2, different dietary Ca/P levels had no significant effect on tibial length, tibial weight, weight per unit length, and density in the experimental Ningxiang pigs (*p* > 0.05). The ash content of the metatarsal bones exhibited a significant effect, initially increasing and then decreasing with rising dietary Ca/P levels (*p* < 0.001), with the peak observed at a Ca/P level of 0.60/0.48% (Figure 3). Conversely, the Ca content within the metatarsal ash showed an inverted U-shaped pattern, first declining and then increasing (*p* = 0.014), and reaching its highest concentration at a Ca/P level of 0.80/0.64%. The P content in the metatarsal ash was not significantly influenced by varying dietary Ca/P levels (*p* > 0.05). Similarly, the Ca/P ratio in the ash displayed a significant quadratic trend, initially decreasing and then increasing (*p* = 0.001), with the lowest value recorded at a Ca/P level of 0.60/0.48%.

### 3.5. Intestinal Morphology

As illustrated in Table 10 and Table 11, the effects of varying dietary Ca/P levels on intestinal morphology were evaluated in Ningxiang pigs during the 50–80 kg growth stage. In the duodenum, changes in Ca/P levels did not significantly affect the villus height (VH), crypt depth (CD), villus surface area, and VH/CD ratio (*p* > 0.05). VH exhibited a quadratic trend (*p* = 0.070), peaking at a Ca/P level of 0.60/0.48%. Villus width (VW) increased linearly with increasing Ca/P levels (*p* = 0.085). In the jejunum, increasing dietary Ca/P levels also had no significant effects on the VW and VH:CD ratio (*p* > 0.05). Jejunal VH showed a quadratic trend (*p* = 0.092), reaching its highest point at a Ca/P level of 0.60/0.48%; while CD displayed a U-shaped trend (*p* = 0.052), with the lowest value observed at the same Ca/P level. Notably, dietary Ca/P levels significantly affected the villus surface area (*p* = 0.029), which was maximized at a Ca/P level of 0.60/0.48%.

### 3.6. Expression of Genes Encoding Intestinal Calcium–Phosphate Transporters

As presented in Table 12, increasing the dietary Ca/P levels during the 50–80 kg growth stage of Ningxiang pigs resulted in a linear increase in the expression of the calcium transporters *CaBP-D9K* and *CaSR* in the duodenum (*p* < 0.05), with peak expression observed at a Ca/P level of 0.80/0.64%. In contrast, no significant differences were detected in the relative expression of *CaBP-D28K* and *TRPV6* (*p* > 0.05). For phosphorus transporters, *SLC20A1* exhibited a significant effect (*p* = 0.022), with the highest expression at a Ca/P level of 0.50/0.40%. No significant differences were observed in the relative expression levels of *SLC34A1*, *SLC34A2*, *SLC34A3*, and *SLC20A1* (*p* > 0.05).

As shown in Table 13, in the jejuna of Ningxiang pigs during the 50–80 kg growth stage, increasing dietary Ca/P levels led to a linear growth trend in the expression of the calcium transporters *CaBP-D9K* and *CaSR* (*p* < 0.10). The relative expression of *CaBP-D28K* and *TRPV6* did not show significant differences (*p* > 0.05). For phosphorus transporters, *SLC34A2* exhibited an increasing and then decreasing trend (*p* = 0.007), peaking at a Ca/P level of 0.60/0.48%. The relative expression of *SLC34A3* decreased significantly (*p* = 0.045), with peak expression at a Ca/P level of 0.50/0.40%. No significant differences were observed in the relative expression levels of *SLC34A1*, *SLC20A1*, and *SLC20A2* (*p* > 0.05).

### 3.7. Results of Gut Microbiota Diversity

#### 3.7.1. α-Diversity Indices and Inter-Group Similarity Analysis

**Figure 4:**
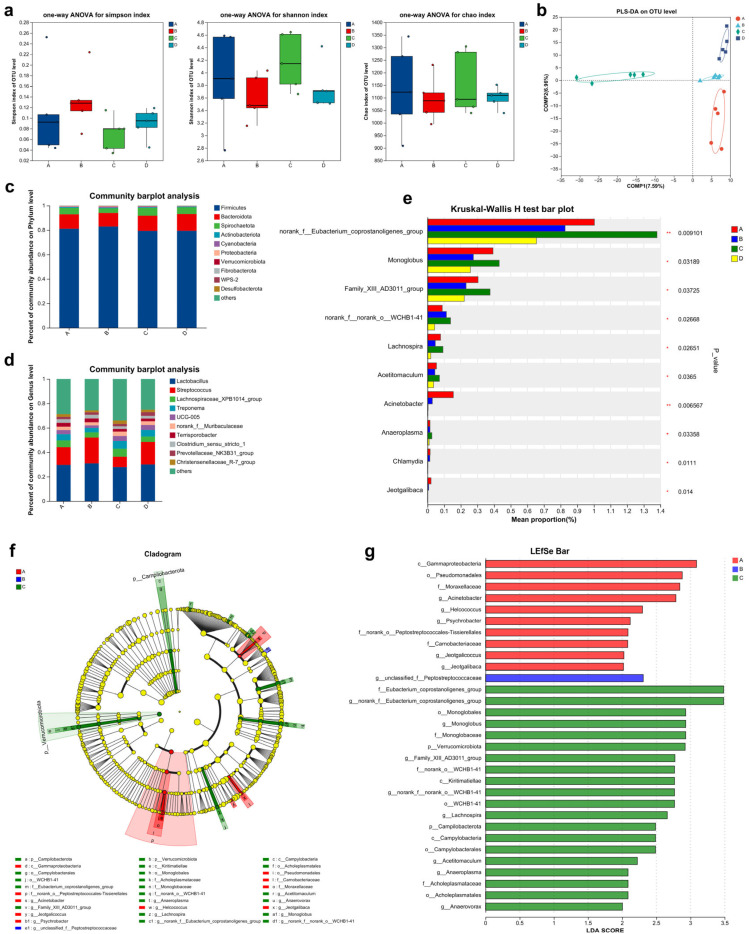
The effects of dietary Ca/P levels on gut microbiota in 50–80 kg Ningxiang pigs (n = 5): (**a**) Alpha diversity index. (**b**) Similarity analysis among experimental groups. (**c**) Community composition at the phylum level. (**d**) Species composition analysis at the genus level. (**e**) Microorganisms with significant differences at the genus level. (**f**) The outcomes of LEfSe multi-level species difference discriminant analysis. (**g**) The LDA values of different differential species. A: Ca/P level = 0.50/0.40%; B: Ca/P level = 0.60/0.48%; C: Ca/P level = 0.70/0.56%; D: Ca/P level = 0.80/0.64%. “p”: phylum, “c”: class, “o”: order, “f”: family, “g”: genus, “s”: species.

The results of α-diversity analysis (Figure 4a) showed that dietary Ca/P levels had no significant effect on the Shannon, Simpson, and Chao1 indices (*p* > 0.05). The PLS-DA scores plot (Figure 4b) demonstrated distinct bacterial community composition among the four treatment groups.

#### 3.7.2. Community Composition Analysis

As presented in Figure 4c,d, at the phylum level, Firmicutes was the dominant phylum across all four groups, representing 81.13%, 82.87%, 79.31%, and 79.39% of the total bacterial count in groups A, B, C, and D, respectively. At the genus level, the top five dominant genera by average relative abundance in each group were as follows: Group A: *Lactobacillus* (29.59%), *Streptococcus* (14.55%), *Lachnospiraceae_XPB1014* (5.51%), *Treponema* (5.16%), and *UCG-005* (3.32%); Group B: *Lactobacillus* (30.86%), *Streptococcus* (21.17%), *Lachnospiraceae_XPB1014* (4.22%), *Treponema* (3.64%), and *UCG-005* (2.07%); Group C: *Lactobacillus* (27.89%), *Streptococcus* (8.38%), *Lachnospiraceae_XPB1014* (6.52%), *Treponema* (6.36%), and *UCG-005* (4.17%); Group D: *Lactobacillus* (29.90%), *Streptococcus* (18.59%), *Lachnospiraceae_XPB1014* (4.37%), *Treponema* (5.40%), and *UCG-005* (4.00%). Furthermore, as shown in Figure 4e, species differences analysis revealed that the relative abundance of *Acinetobacter* (*p* < 0.01), *Chlamydia*, and *Jeotgalibaca* was highest in Group A (*p* < 0.05). In contrast, Group C exhibited the highest relative abundance of *norank_f_ Eubacterium_coprostanoligenes_group* (*p* < 0.01), *Monoglobus*, *Family_XIII_AD3011_group*, *norank_f_norank_o_WCHB1-41*, *Lachnospira*, *Acetitomaculum*, and *Anaeroplasma* (*p* < 0.05).

#### 3.7.3. LEfSe Analysis

The LEfSe analysis (LDA > 2) identified significant differences in the relative abundance of taxa at various taxonomic levels among groups A–D (Figure 4f,g). In Group A, the key biomarkers included *Gammaproteobacteria*, *Pseudomonadales*, *Moraxellaceae*, *Acinetobacter*, *Helcococcus*, *Psychrobacter*, *norank_o_Peptostreptococcales-Tissierellales*, *Carnobacteriaceae*, *Jeotgalicoccus*, and *Jeotgalibaca*. Conversely, Group B was characterized by *g_unclassified_f_Peptostreptococcaceae*. For Group C, the biomarkers encompassed *Eubacterium_coprostanoligenes_group*, *norank_f_Eubacterium_coprostanoligenes_group*, *Monoglobales*, *Monoglobus*, *Monoglobaceae*, *Verrucomicrobiota*, *Family_XIII_AD3011_group*, *norank_o_WCHB1-41*, *Kiritimatiellae*, *norank_f_norank_o_WCHB1-41*, *o_WCHB1-41*, *Lachnospira*, *Campilobacterota*, *Campylobacteria*, *Campylobacterales*, *Acetitomaculum*, *Anaeroplasma*, *Acholeplasmataceae*, *Acholeplasmatales*, and *Anaerovorax*.

## 4. Discussion

While Ca/P requirements in commercial hybrids are well documented, data remain scarce for slow-growing, adipose-predisposed breeds like Ningxiang pigs. Characterized by delayed maturity and elevated adiposity, Ningxiang pigs exhibit distinct Ca/P requirements that diverge from those of lean-type pig breeds. These findings establish stage-specific mineral requirements in this breed, challenging the conventional Ca/P levels established for lean-type genotypes.

During the 30–50 kg phase, 0.83/0.67% Ca/P maximized ADG and ADFI, whereas 0.60/0.48% optimized growth efficiency in the 50–80 kg phase. This inverse relationship in later stages aligns with reports that excess mineral supplementation impairs nutrient utilization in finishing pigs [22]. Critically, divergent Ca/P optima for growth (0.60/0.48%) versus bone mineralization (0.80/0.64%) were observed, revealing a key nutrient partitioning dynamic in this breed.

Blood biochemical indices serve as crucial indicators of metabolic nutrients in livestock. Variations in serum Ca and P levels significantly influence regulatory hormones affecting skeletal development. Previous research demonstrates that serum Ca/P concentrations are tightly regulated through endocrine mechanisms to maintain homeostasis [23]. In the present study, serum Ca and P levels showed no significant differences between growth stages, although higher values occurred during rapid bone development (30–50 kg phase). Serum Ca regulation involves widely distributed calcium-sensing receptors, while phosphorus sensing is primarily confined to the intestine and parathyroid glands, resulting in less stringent P regulation [24]. As Su et al. [25] reported, serum Ca/P levels remain unaffected by dietary calcium concentrations within requirement thresholds.

Non-esterified fatty acid (NEFA) function as lipid metabolism intermediates. Serum NEFA and triglyceride trends indicate that dietary Ca/P modulation influences lipid metabolism. Specifically, the 0.60/0.48% Ca/P level promoted favorable fat deposition patterns, potentially through calcitriol-mediated mechanisms, where low-calcium diets increase calcitriol activity, stimulating lipogenesis while inhibiting lipolysis [26]. At 0.80/0.64% Ca/P, serum NEFA levels were significantly elevated, while triglycerides peaked at 0.60/0.48%. These findings demonstrate that dietary Ca/P variations impact lipid metabolism in Ningxiang pigs, with 0.60/0.48% facilitating optimal fat accumulation and weight gain. Current research on Ca/P–lipid metabolism interactions remains limited, warranting further mechanistic investigation, particularly for indigenous swine genotypes.

Dietary Ca/P levels profoundly impact skeletal development, since these minerals are predominantly stored in bone tissue [27]. In this study, the metatarsal ash content peaked at 0.60/0.48% Ca/P, while the maximal tibia weight and bone Ca content occurred at 0.80/0.64%. This indicates that the Ca/P requirements for bone mineralization exceed those for growth performance optimization (0.60/0.48%), emphasizing the need for phase-specific strategies to balance skeletal development and growth efficiency. These results corroborate previous observations that P deficiency disproportionately reduces bone ash deposition [28,29], although Ningxiang pigs exhibit greater resilience to moderate Ca/P reductions than commercial breeds.

During the 30–50 kg growth phase, maximal energy and P digestibility occurred at 0.83/0.67% Ca/P. In the 50–80 kg phase, 0.60/0.48% Ca/P yielded the highest energy digestibility, with CP and Ca digestibility second only to 0.70/0.56%. This suggests that excessive Ca/P concentrations may hinder nutrient absorption. Optimal levels improved the small intestine morphology, facilitating nutrient absorption and growth [17]. Intestinal villi play a key role in nutrient absorption. Shorter villi and deeper crypts reduce absorption and increase secretion due to fewer absorptive cells and more secretory cells [30,31]. At 50–80 kg, with a 0.60/0.48% Ca/P level, the duodenal villi and surface area maximized, jejunal villi height peaked, crypts shallowed, and villus surface area increased. These morphological enhancements correspond with improved nutrient digestibility and superior growth performance.

Calcium absorption in the small intestine maintains homeostasis through transcellular and paracellular pathways [32,33]. Transcellular absorption dominates with low calcium intake, requiring TRPV6 channels, calbindin proteins, and energy [34,35]. Paracellular absorption increases with adequate/high intake, and TRPV6-mediated transport is inhibited by calcium-sensing receptor (CaSR) activation [36,37]. Dietary Ca concentration regulates transporter expression in Ningxiang pigs. During finishing (50–80 kg), duodenal and jejunal *CaBP-D9K* expression increased with dietary Ca, while *CaBP-D28K* expression peaked then declined at 0.80/0.64% Ca. *CaSR* expression significantly increased at 0.70/0.56% and 0.80/0.64% versus lower levels. These results indicate that Ca absorption saturation occurs between 0.7 and 0.8% dietary Ca during finishing, explaining growth inhibition at higher levels through calcium–phosphorus complex formation [38].

Dietary Ca/P variations significantly influenced the gut microbiota composition. Firmicutes dominated across treatments, with maximal abundance at 0.60/0.48% Ca/P, facilitating food digestion and microecological balance [5]. *Lactobacillus* abundance increased at this level, consistent with its probiotic functions [39,40]. Similarly, *Streptococcus* abundance rose, supporting its roles in digestion and vitamin synthesis [41]. Notably, the relative abundance of *Peptostreptococcaceae* significantly increased at 0.60/0.48% Ca/P. This is functionally important, as *Peptostreptococcaceae* produce butyrate [42], which enhances mineral absorption [43], inhibits osteoclast differentiation and bone resorption [44], and maintains intestinal barrier integrity [45,46]. These findings demonstrate that optimal Ca/P levels promote beneficial microbial communities that support intestinal health and growth efficiency [6].

## 5. Conclusions

This study determined stage-specific Ca/P requirements for Ningxiang pigs. During the growth phase (30–50 kg), a dietary Ca/P level of 0.83%/0.67% was demonstrated to maximize growth performance and enhance energy and phosphorus utilization. In the fattening phase (50–80 kg), a Ca/P level of 0.60%/0.48% optimized ADG and feed efficiency by improving the intestinal morphology, upregulating the expression of Ca/P transporters (*CaSR*, *SLC34A2*), and modulating the gut microbiota favorably. Notably, the Ca/P levels that optimized growth were 25–29% lower than those required for optimal bone mineralization (0.80%/0.64%), underscoring the nutrient partitioning priorities that favor mobility over skeletal deposition. Additionally, while regulation of the gut microbiota was observed, the causal mechanisms linking *Peptostreptococcaceae* enrichment to mineral absorption efficiency require further microbiome and metabolomics studies. These findings provide a comprehensive nutritional framework for optimizing growth performance, maintaining skeletal integrity, and promoting microbial homeostasis in indigenous swine breeds.

Based on these findings, it is recommended that farmers and feed formulators for Ningxiang pigs adopt a two-phase feeding strategy specifically tailored to Ca/P levels: Growth Phase (30–50 kg)—Utilize diets containing 0.83% Ca and 0.67% P to maximize growth rate and feed efficiency. Fattening Phase (50–80 kg)—Transition to diets containing 0.60% Ca and 0.48% P to sustain optimal growth performance and feed conversion while potentially reducing feed costs associated with mineral supplementation.

## Figures and Tables

**Table 1:** Feed ingredients and nutritional composition (air-dried basis) for the growth phase (30–50 kg).

Items	Groups			
	A1	B1	C1	D1
Corn, %	55.49	55.17	54.65	54.2
Wheat middlings, %	15	15	15	15
Wheat bran, %	6.7	6.7	6.7	6.7
43% Rice bran, %	9	9	9	9
Broken rice, %	10	10	10	10
Stone powder, %	1.065	1.275	1.255	1.245
CaHPO_4_, %	0	0.11	0.65	1.11
Salt, %	0.4	0.4	0.4	0.4
Premix ^1^, %	2	2	2	2
Antifungal, %	0.05	0.05	0.05	0.05
70 L-lysine sulfate, %	0.255	0.255	0.255	0.255
Threonine, %	0.04	0.04	0.04	0.04
Total, %	100	100	100	100
Nutritional level (calculated)				
NE ^3^, MJ/kg	9.42	9.42	9.42	9.42
CP ^4^, %	13	13	13	13
Ca, %	0.52	0.62	0.73	0.83
Total P, %	0.48	0.50	0.59	0.67
Available P, %	0.15	0.17	0.26	0.34
SID Lys ^2^, %	0.6	0.6	0.6	0.6
SID Met, %	0.19	0.19	0.19	0.19
SID M + C, %	0.38	0.38	0.38	0.38
SID Thr, %	0.41	0.41	0.41	0.41
SID Trp, %	0.12	0.12	0.12	0.12
SID Val, %	0.51	0.51	0.51	0.51
Nutritional level (measured)				
CP, %	13.38	15.00	15.57	14.56
Ca, %	0.54	0.59	0.73	0.85
Total P, %	0.57	0.57	0.65	0.72

^1^ Vitamins and minerals per kilogram of diet: 0.2% potassium, 0.05% magnesium, 70 mg iron, 4 mg copper, 3 mg manganese, 55 mg zinc, 0.15 mg iodine, 0.25 mg selenium, 1700 IU vitamin A, 200 IU vitamin D3, 20 IU vitamin E, 0.5 mg vitamin K, 1.5 mg thiamine, 8 μg vitamin B12, 2.5 mg riboflavin, 9 mg D-pantothenic acid, 10 mg niacin, 1 mg pyridoxine, 0.1 mg biotin, 0.3 mg folic acid, 0.40 g choline. ^2^ SID, standard ileal digestibility. ^3^ NE, net energy. ^4^ CP, crude protein.

**Table 2:** Feed ingredients and nutritional composition (air-dried basis) for the fattening phase (50–80 kg).

Items	Groups			
	A	B	C	D
Corn, %	53	53	53	53
Wheat middlings, %	20	20	20	20
Wheat bran, %	10	10	10	10
43% Rice bran, %	5.7	5.7	5.7	5.7
Broken rice, %	6	6	6	6
Stone powder, %	1.2	1.38	1.37	1.35
CaHPO_4_, %	0	0.16	0.64	1.1
Salt, %	0.4	0.4	0.4	0.4
Premix ^1^, %	2	2	2	2
Antifungal, %	0.05	0.05	0.05	0.05
70 L-lysine sulfate, %	0.25	0.25	0.25	0.25
Threonine, %	0.03	0.03	0.03	0.03
Total, %	100	100	100	100
Nutritional level (calculated)				
NE ^3^, MJ/kg	9.28	9.28	9.28	9.28
CP ^4^, %	12	12	12	12
Ca, %	0.5	0.6	0.7	0.8
Total P, %	0.45	0.48	0.56	0.64
Available P, %	0.14	0.17	0.25	0.33
SID Lys ^2^, %	0.52	0.52	0.52	0.52
SID Met, %	0.14	0.14	0.14	0.14
SID M + C, %	0.32	0.32	0.32	0.32
SID Thr, %	0.33	0.33	0.33	0.33
SID Trp, %	0.11	0.11	0.11	0.11
SID Val, %	0.45	0.45	0.45	0.45
SID Lle, %	0.35	0.35	0.35	0.35
Nutritional level (measured)				
CP, %	14.32	14.31	14.35	14.30
Ca, %	0.61	0.83	0.89	0.92
Total P, %	0.53	0.56	0.62	0.74

^1^ Vitamins and minerals per kilogram of diet: 0.2% potassium, 0.05% magnesium, 90 mg iron, 4 mg copper, 3 mg manganese, 65 mg zinc, 0.15 mg iodine, 0.25 mg selenium, 2100 IU vitamin A, 225 IU vitamin D3, 20 IU vitamin E, 0.8 mg vitamin K, 1.5 mg thiamine, 15 μg vitamin B12, 3.5 mg riboflavin, 10.5 mg D-pantothenic acid, 15 mg niacin, 1 mg pyridoxine, 0.1 mg biotin, 0.5 mg folic acid, 0.45 g choline. ^2^ SID, standard ileal digestibility. ^3^ NE, net energy. ^4^ CP, crude protein.

**Table 3:** Information on Ca and P transporter-related genes and their qPCR primers.

Gene	Primer	Primer Sequence (5′-3′)	Size (bp)	GenBank Accession No.
*CaBP-D9K*	Forward	CAGCCAAAGAAGGGGATCCAAACC	102	NM_214040.2
	Reverse	CATCTAGGGTTCTCGGACCTTTCAG		
*CaBP-D28K*	Forward	ACGATCAGGATGGCAATGGAT	156	NM_001130226.1
	Reverse	TTCGGTACAGCTTCCCTCCA		
*CaSR*	Forward	CAGAGACTGTTGGCGGAGTC	257	XM_021068447.1
	Reverse	CGACTTCCCAGGATGAGGTG		
*TRPV6*	Forward	CACTTTAGGAGAGGCTTGCTG	147	XM_021078887.1
	Reverse	ATGACTTTATTGGAAGGTAGGAGGT		
*SLC34A1*	Forward	CTGATGCTTGCCTTCCTCTACCTC	111	XM_021082348.1
	Reverse	GATGGCGTTGTCCTTGAAGATGTC		
*SLC34A2*	Forward	CACAAAGATGACACCGGCAC	124	XM_005666562.3
	Reverse	TGTTCTTAAGCTCGGGCAGG		
*SLC34A3*	Forward	GCATCCTGTTGTGGTACGTG	120	XM_021081188.1
	Reverse	AGAAGCCGAGCAACAGGTAG		
*SLC20A1*	Forward	GATGTTTGGTTCTGCTGTGTG	133	XM_021087022.1
	Reverse	AGACCACTTGACACCCTCCTG		
*SLC20A2*	Forward	CCAGGTGCCAAGGCTAATGA	238	XM_021078067.1
	Reverse	AGAGCCCTGAATCCTTGTGC		
*RPL4*	Forward	CGCTGGTCATGTCTAAAGGTCA	202	XM_005659862.3
	Reverse	ATTCGGCGACGGTTTCTCAT		

**Table 4:** Effects of dietary Ca/P levels on growth performance in 30–50 kg Ningxiang pigs (n = 5).

Items	Groups				SEM	*p*-Value		
	A1	B1	C1	D1		Overall	Linear	Quadratic
BW_initial_ (kg)	32.37	32.39	32.10	32.26	0.40	0.995	0.875	0.937
BW_final_ (kg)	48.53	49.24	48.72	51.95	0.65	0.218	0.097	0.324
ADG (kg/d)	0.37	0.38	0.37	0.44	0.01	0.158	0.076	0.236
ADFI (kg/d)	1.39 ^ab^	1.37 ^b^	1.48 ^ab^	1.60 ^a^	0.04	0.173	0.047	0.376
F:G	3.79	3.61	3.95	3.66	0.09	0.551	0.982	0.762

^a,b^ Within the same row, means not sharing a common superscript letter are significantly different. ADG: average daily weight gain. ADFI: average daily feed intake. F:G: feed-to-gain ratio. BW_initial_: initial body weight. BW_final_: final body weight. A1: Ca/P level = 0.52/0.42; B1: Ca/P level = 0.62/0.50; C1: Ca/P level = 0.73/0.59; D1: Ca/P level = 0.83/0.67.

**Table 5:** Effects of dietary Ca/P levels on growth performance in 50–80 kg Ningxiang pigs (n = 5).

Items	Groups				SEM	*p*-Value		
	A	B	C	D		Overall	Linear	Quadratic
BW_initial_ (kg)	50.87	51.01	50.88	50.97	0.56	1.000	0.976	0.984
BW_final_ (kg)	79.51	81.60	78.24	79.54	0.89	0.639	0.695	0.831
ADG (kg/d)	0.51 ^ab^	0.54 ^a^	0.47 ^b^	0.49 ^b^	0.01	0.021	0.095	0.844
ADFI (kg/d)	2.13	2.25	2.05	2.11	0.32	0.146	0.371	0.633
F:G	4.20	4.18	4.36	4.27	0.37	0.315	0.223	0.632

^a,b^ Within the same row, means not sharing a common superscript letter are significantly different. ADG: average daily weight gain. ADFI: average daily feed intake. F:G: feed-to-gain ratio. BW_initial_: initial body weight. BW_final_: final body weight. A: Ca/P level = 0.50/0.40; B: Ca/P level = 0.60/0.48; C: Ca/P level = 0.70/0.56; D: Ca/P level = 0.80/0.64.

**Table 6:** Effects of dietary Ca/P levels on blood biochemical parameters in 30–50 kg Ningxiang pigs (n = 8).

Items	Groups				SEM	*p*-Value		
	A1	B1	C1	D1		Overall	Linear	Quadratic
ALP (U/L)	207.38	271.67	228.29	219.00	15.53	0.468	0.952	0.251
BUN (mmol/L)	6.08	6.29	5.81	6.66	0.33	0.849	0.674	0.644
Ca (mmol/L)	3.37	3.49	3.59	3.50	0.03	0.187	0.111	0.131
P (mmol/L)	4.34	4.36	4.20	4.66	0.10	0.439	0.381	0.271
TG (μg/L)	0.86	0.84	0.78	0.90	0.05	0.868	0.884	0.472
CHOL (mg/dL)	3.97	3.82	3.61	3.47	0.12	0.497	0.13	0.968
NEFA (mmol/L)	0.17	0.26	0.17	0.21	0.03	0.531	0.893	0.675

ALP: alkaline phosphatase, BUN: blood urea nitrogen, Ca: serum calcium, P: inorganic phosphorus, TG: triglycerides, CHOL: total cholesterol, NEFA: non-esterified fatty acid. A1: Ca/P level = 0.52/0.42%; B1: Ca/P level = 0.62/0.50%; C1: Ca/P level = 0.73/0.59%; D1: Ca/P level = 0.83/0.67%.

**Table 7:** Effects of dietary Ca/P levels on blood biochemical parameters in 50–80 kg Ningxiang pigs (n = 8).

Items	Groups				SEM	*p*-Value		
	A	B	C	D		Overall	Linear	Quadratic
ALP (U/L)	179.00	178.71	160.88	147.63	9.04	0.568	0.179	0.727
BUN (mmol/L)	2.69	3.00	2.84	2.79	0.09	0.677	0.864	0.325
Ca (mmol/L)	2.72	2.83	2.77	2.71	0.02	0.118	0.063	0.304
P (mmol/L)	3.23	3.14	3.01	3.12	0.05	0.387	0.285	0.279
TG (μg/L)	0.44	0.62	0.48	0.52	0.03	0.162	0.086	0.057
CHOL (mg/dL)	2.72	3.14	2.74	2.64	0.09	0.205	0.141	0.161
NEFA (mmol/L)	0.09 ^b^	0.08 ^b^	0.09 ^b^	0.15 ^a^	0.01	0.083	0.046	0.127

^a,b^ Within the same row, means not sharing a common superscript letter are significantly different. ALP: alkaline phosphatase, BUN: blood urea nitrogen, Ca: serum calcium, P: inorganic phosphorus, TG: triglycerides, CHOL: total cholesterol, NEFA: non-esterified fatty acid. A: Ca/P level = 0.50/0.40%; B: Ca/P level = 0.60/0.48%; C: Ca/P level = 0.70/0.56%; D: Ca/P level = 0.80/0.64%.

**Table 8:** Effects of dietary Ca/P levels on apparent nutrient digestibility in 30–50 kg Ningxiang pigs (n = 8).

Items	Groups				SEM	*p*-Value		
	A1	B1	C1	D1		Overall	Linear	Quadratic
ATTD GE (%)	83.97 ^ab^	81.69 ^b^	81.77 ^b^	85.63 ^a^	0.61	0.044	0.291	0.009
ATTD CP (%)	82.86	82.96	80.58	81.80	0.59	0.443	0.318	0.64
ATTD Ca (%)	43.04	28.63	34.70	45.02	2.92	0.175	0.104	0.515
ATTD P (%)	37.17 ^ab^	23.56 ^b^	33.66 ^ab^	44.23 ^a^	2.91	0.072	0.191	0.031

^a,b^ Within the same row, means not sharing a same superscript letter are significantly different. ATTD: apparent total tract digestibility. CP: crude protein. GE: gross energy. A1: Ca/P level = 0.52/0.42%; B1: Ca/P level = 0.62/0.50%; C1: Ca/P level = 0.73/0.59%; D1: Ca/P level = 0.83/0.67%.

**Table 9:** Effects of dietary Ca/P levels on apparent nutrient digestibility in 50–80 kg Ningxiang pigs (n = 8).

Items	Groups				SEM	*p*-Value		
	A	B	C	D		Overall	Linear	Quadratic
ATTD GE (%)	73.93 ^b^	82.48 ^a^	81.86 ^a^	78.75 ^ab^	1.09	0.009	0.082	0.003
ATTD CP (%)	75.98 ^b^	82.37 ^a^	82.74 ^a^	79.20 ^ab^	1.02	0.051	0.221	0.012
ATTD Ca (%)	13.31 ^b^	36.94 ^a^	37.13 ^a^	18.22 ^ab^	3.68	0.019	0.626	0.013
ATTD P (%)	19.50	27.80	31.96	25.86	2.64	0.566	0.495	0.332

^a,b^ Within the same row, means not sharing a common superscript letter are significantly different. ATTD: apparent total tract digestibility. CP: crude protein. GE: gross energy. A: Ca/P level = 0.50/0.40%; B: Ca/P level = 0.60/0.48%; C: Ca/P level = 0.70/0.56%; D: Ca/P level = 0.80/0.64%.

**Table 10:** Effects of dietary Ca/P levels on duodenal morphology in 50–80 kg Ningxiang pigs (n = 8).

Items	Groups				SEM	*p*-Value		
	A	B	C	D		Overall	Linear	Quadratic
Villus height (μm)	330.49	400.20	367.35	347.20	13.02	0.206	0.874	0.070
Crypt depth (μm)	508.42	528.51	498.45	531.87	13.23	0.805	0.656	0.379
Villus width (μm)	147.30	152.10	159.91	164.27	3.88	0.376	0.085	0.977
VH/CD	0.67	0.74	0.73	0.69	0.03	0.866	0.835	0.423
Villus surface area (μm^2^)	154,468.12	195,552.06	184,829.87	176,956.40	9197.68	0.449	0.506	0.204

VH: villus height; CD: crypt depth; VW: villus width. A: Ca/P level = 0.50/0.40%; B: Ca/P level = 0.60/0.48%; C: Ca/P level = 0.70/0.56%; D: Ca/P level = 0.80/0.64%.

**Table 11:** Effects of dietary Ca/P levels on jejunal morphology in 50–80 kg Ningxiang pigs (n = 8).

Items	Groups				SEM	*p*-Value		
	A	B	C	D		Overall	Linear	Quadratic
Villus height (μm)	578.24	670.85	631.44	655.08	13.80	0.092	0.12	0.194
Crypt depth (μm)	1054.47	1007.42	1155.32	1118.84	21.24	0.052	0.064	0.893
Villus width (μm)	394.79	428.13	417.66	425.31	7.80	0.467	0.265	0.421
VH/CD	0.60	0.67	0.55	0.62	0.02	0.232	0.809	0.988
Villus surface area (μm^2^)	730,813.42 ^b^	900,145.88 ^a^	823,537.06 ^ab^	894,183.62 ^a^	23,724.99	0.029	0.035	0.219

^a,b^ Within the same row, means not sharing a common superscript letter are significantly different. VH: villus height; CD: crypt depth; VW: villus width. A: Ca/P level = 0.50/0.40%; B: Ca/P level = 0.60/0.48%; C: Ca/P level = 0.70/0.56%; D: Ca/P level = 0.80/0.64%.

**Table 12:** Effects of dietary Ca/P levels on duodenal mRNA expression of calcium–phosphate transporter-related genes in 50–80 kg Ningxiang pigs (n = 8).

Items	Groups				SEM	*p*-Value		
	A	B	C	D		Overall	Linear	Quadratic
*CaBP-D9K*	1.06 ^b^	1.53 ^ab^	1.67 ^ab^	1.82 ^a^	0.12	0.084	0.015	0.468
*CaSR*	1.11 ^b^	2.83 ^ab^	7.02^ab^	8.19 ^a^	1.09	0.05	0.007	0.892
*CaBP-D28K*	0.74	0.75	0.91	0.82	0.08	0.897	0.591	0.765
*TRPV6*	1.03	1.14	1.25	1.02	0.13	0.932	0.955	0.541
*SLC34A1*	0.68	0.39	1.00	0.76	0.12	0.378	0.432	0.93
*SLC34A2*	0.96	1.98	1.30	2.10	0.27	0.372	0.252	0.835
*SLC34A3*	1.44	0.82	0.85	1.10	0.15	0.419	0.452	0.151
*SLC20A1*	1.59 ^a^	1.15 ^ab^	0.62 ^b^	1.14 ^ab^	0.11	0.022	0.022	0.032
*SLC20A2*	1.09	0.71	1.25	1.06	0.27	0.228	0.589	0.62

^a,b^ Within the same row, means not sharing a common superscript letter are significantly different. A: Ca/P level = 0.50/0.40%; B: Ca/P level = 0.60/0.48%; C: Ca/P level = 0.70/0.56%; D: Ca/P level = 0.80/0.64%.

**Table 13:** Effects of dietary Ca/P levels on jejunal mRNA expression of calcium–phosphate transporter-related genes in 50–80 kg Ningxiang pigs (n = 8).

Items	Groups				SEM	*p*-Value		
	A	B	C	D		Overall	Linear	Quadratic
*CaBP-D9K*	0.72	0.98	1.07	1.29	0.11	0.325	0.073	0.913
*CaSR*	1.68	2.56	1.50	1.03	0.21	0.061	0.09	0.093
*CaBP-D28K*	0.76	1.02	1.06	0.89	0.08	0.526	0.53	0.186
*TRPV6*	1.05	1.32	1.30	1.41	0.10	0.603	0.184	0.956
*SLC34A1*	2.22	1.26	0.90	1.38	0.25	0.304	0.212	0.162
*SLC34A2*	1.10 ^b^	2.83 ^a^	1.65 ^b^	1.63 ^b^	0.19	0.007	0.788	0.013
*SLC34A3*	1.12 ^a^	0.80 ^ab^	1.05 ^a^	0.54 ^b^	0.08	0.045	0.135	0.059
*SLC20A1*	1.37	1.50	1.06	1.28	0.10	0.492	0.434	0.809
*SLC20A2*	1.33	1.16	0.93	0.98	0.10	0.512	0.799	0.71

^a,b^ Within the same row, means not sharing a common superscript letter are significantly different. A: Ca/P level = 0.50/0.40%; B: Ca/P level = 0.60/0.48%; C: Ca/P level = 0.70/0.56%; D: Ca/P level = 0.80/0.64%.

## Data Availability

The data from this study have not been deposited in an official repository. The authors are willing to share the information upon reasonable request.

## Abbreviations

VH	Villus height
CD	Crypt depth
ADG	Average daily gain
ADFI	Average daily feed intake
F:G	Feed-to-gain ratio
BWinitial	Initial body weight
BWfinal	Final body weight
ATTD	Apparent total tract digestibility
GE	Gross energy
SEM	Standard error of the mean
TW	Tibia weight
TL	Tibia length
BMD	Bone mineral density
CP	Crude protein
